# Proposal of a Simpler Eye‐Level Risk Model Incorporating Reticular Pseudodrusen for the Clinical Prediction of Late Age‐Related Macular Degeneration

**DOI:** 10.1111/ceo.14576

**Published:** 2025-07-03

**Authors:** Matt Trinh, Annita Duong, Rene Cheung, Simon Chen, David Ng, Jeff Friedrich, Chris Hodge, Lisa Nivison‐Smith, Angelica Ly

**Affiliations:** ^1^ School of Optometry and Vision Science University of New South Wales Sydney New South Wales Australia; ^2^ Vision Eye Institute Sydney New South Wales Australia

**Keywords:** age‐related macular degeneration, clinical, prediction, prognosis, reticular pseudodrusen, risk

## Abstract

**Background:**

The updated simplified AREDS risk model predicts progression to late age‐related macular degeneration (AMD) by person, describing up to nine observations across both eyes and 10 annual risk scores (0–4, with/without reticular pseudodrusen [RPD]). This study proposes an abridged model to enable inter‐eye comparisons and potentially enhance clinical efficiency.

**Methods:**

This retrospective cohort study included 269 participants with early/intermediate AMD over 7 years. The full, person‐level updated simplified AREDS risk model was compared to eye‐level candidate risk models, derived by removing the least predictive biomarkers. The main outcomes were prognostic performance (AUC) and risk score separability (*χ* ^2^).

**Results:**

At 1–3 years, the full model showed prognostic performance (AUC ± SE) up to 84.52% ± 5.93%, with overlap between most risk scores (*χ* ^2^ ≤ 2.08). Removing large drusen and pigmentary abnormalities in the fellow eye, intermediate drusen in both eyes, and redefining RPD presence as eye‐specific maintained prognostic performance (up to 84.71% ± 4.72%). Assigning one point per retained biomarker, based on similar adjusted risks, improved risk score separability (*χ* ^2^ ≥ 3.85, *p* < 0.05) while reducing the number of annual scores from 10 to five.

**Conclusions:**

The updated simplified AREDS risk model can be essentially halved without compromising prognostic performance by deriving eye‐specific biomarkers and assigning one point per biomarker (large drusen, pigmentary abnormalities, and RPD in the primary eye, and late AMD in the fellow eye). This eye‐level risk stratification may improve clinical efficiency and inter‐eye study designs when one eye is of particular interest. An example of 3‐year risks (scores 0–4) was ≈4%, 8%, 16%, 32%, and 64%.

## 1 Introduction

Early and accurate prediction of age‐related macular degeneration (AMD) progression, particularly the onset of late AMD, is crucial for facilitating timely interventions that may reduce its impact on vision, quality‐of‐life, and healthcare systems [1, 2]. The simplified age‐related eye disease study (AREDS) risk model is widely adopted for this purpose [3, 4, 5, 6], providing clear risk categories based on retinal imaging [7] for convenient use in clinical practice and serving as a reference person‐level risk model in research. Recently, an updated simplified AREDS risk scale was released, introducing reticular pseudodrusen (RPD) and modernising the definition of geographic atrophy to include non‐central atrophy [8], albeit increasing the complexity of clinical prognostication which may limit its efficiency and usability.

The updated model now includes up to nine observations across both eyes: (1) large drusen in the primary eye, (2) pigmentary abnormalities in the primary eye, (3) large drusen in the fellow eye (if late AMD is not present), (4) pigmentary abnormalities in the fellow eye (if late AMD is not present), (5) late AMD in the fellow eye, (6, 7) intermediate drusen in both eyes (if large drusen are not present), and (8, 9) RPD in either eye. However, AMD presents asymmetrically in approximately half of cases [9], meaning one eye is often at significantly greater risk than the other. In clinical practice, an eye‐level risk scale may be more practical than a person‐level model, particularly when the fellow eye already has severe vision loss from AMD or otherwise. In clinical studies, an eye‐level approach enables direct assessment of risk between eyes, which is useful for inter‐eye risk‐adjusted study designs, such as randomising interventions at the eye‐level, adaptive trial designs [10], or simplifying pseudo‐random matching of risk between groups (e.g., propensity‐score matching).

Additionally, the inclusion of RPD as a discrete binary factor has doubled the number of risk categories from five (scores 0–4) to 10 (scores 0–4, with [+] or without [−] RPD in either eye). This approach was initially justified by differences in RPD‐associated risk relative to other biomarkers in the model [8]. Using adjusted hazard ratio modelling to account for the co‐presence of biomarkers, rather than evaluating individual biomarker risks in isolation, could clarify whether RPD‐associated risk is comparable to other AREDS biomarkers and hence be reasonably integrated into the scoring system. This would reduce inefficiencies from excessive risk categories, particularly given the substantial overlap in the current model's 10 risk scores, e.g., similar risks between the scores 2 + RPD and 3 − RPD, and 3 + RPD and 4 − RPD.

We hypothesise that deriving an abridged updated simplified AREDS scoring system could streamline its use. This can be achieved by taking an eye‐level approach to reduce the number of observations, and assigning RPD a specific score to reduce the number of annual risk categories. These refinements would improve ease‐of‐use for clinical risk stratification particularly when one AMD eye is of more interest than the other, while also supporting statistical considerations for inter‐eye risk in research study designs.

## 2 Methods

### 2.1 Study Population

The study population consisted of consecutive patients sampled between 2 November 2016 to 10 December 2023 from Vision Eye Institute, a private ophthalmology clinic with three sites in Sydney, Australia (Bondi Junction, Chatswood, and Drummoyne). A fixed seven‐year sampling window was applied with the intention of capturing follow‐up data spanning approximately 5 years, consistent with the follow‐up period used in the original AREDS. However, due to variation in patient retention and review intervals in routine clinical care, analyses focused on outcomes at a mean follow‐up of 3 years. Although the final cohort size was smaller than anticipated, this time frame supported statistically significant models of short‐ to mid‐term risk of progression to late AMD.

All participants were under the care of a senior retinal ophthalmologist (SC). These patients had provided written informed consent for research use of their clinical data, in accordance with the tenets of the Declaration of Helsinki and approved by the Biomedical Human Research Ethics Advisory Committee of the University of New South Wales, Sydney, Australia.

### 2.2 Inclusion and Exclusion Criteria

**FIGURE 1:**
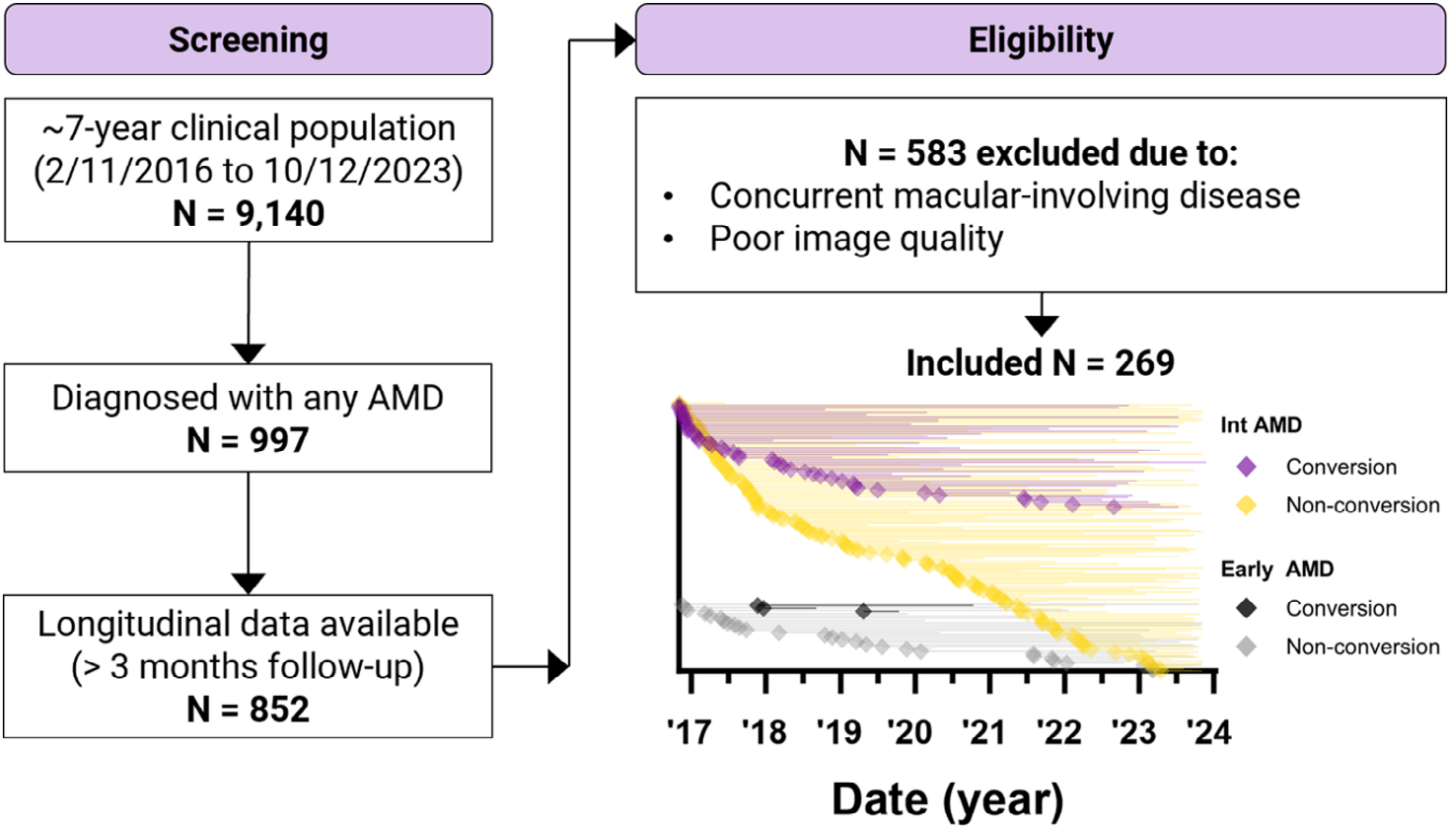
Study population selection. The univariate graph depicts the open cohort sampling of participants with early and intermediate AMD, and whether the study eyes underwent conversion to late AMD over 7‐years. Participant data were censored at the point of conversion to late AMD or after their last visit within the study period. Symbols denote the initial visit and adjacent lines denote the follow‐up time within the study period.

From a total of 9140 screened patients, 997 were identified with any severity of AMD based on clinical records. Of these, 852 had longitudinal data/retinal imaging with > 3 months follow‐up available (Figure 1). The ≥ 3‐month threshold was chosen to exclude individuals lost immediately to follow‐up, while retaining those who required short‐interval review. Although the Royal Australian and New Zealand College of Ophthalmologists (RANZCO) recommends six‐monthly reviews for higher‐risk AMD cases [11], internal audit data from the clinic confirmed that a 3‐month interval was occasionally used for very high‐risk patients. Therefore, this criterion allowed for the inclusion of early converters and detection of transient biomarker changes immediately preceding progression to late AMD.

Another 583 patients were excluded based on the concurrent presence or suspicion of non‐AMD macular‐involving disease, such as optic neuropathy or pachychoroid disease, or poor image quality, i.e., OCT scans with signal strength < 6/10 or significant artefacts. Patients with at least one instance of early or intermediate AMD in either eye during the study period and meeting the above criteria were included. Early and intermediate AMD was confirmed by senior retinal ophthalmologist SC and optometrists MT and AD according to the Beckman Initiative [12]. Specifically, early AMD was defined by the presence of macular medium drusen (≥ 63–125 μm) without pigmentary abnormalities, and intermediate AMD was defined by the presence of macular large drusen (> 125 μm) and/or pigmentary abnormalities with at least medium drusen.

### 2.3 Identification of Imaging Biomarkers

Both eyes per individual were evaluated for imaging biomarkers from the updated simplified AREDS risk model, defined within the macular region two disc‐diameters from the centre. The first eye to progress to late AMD within the study period was designated as the ‘primary’ (1st) eye; if neither eye progressed, one was randomly selected. The other eye was designated as the fellow (2nd) eye, which in some instances exhibited pre‐existing late AMD.

Observations of biomarkers included large drusen in the primary eye, pigmentary abnormalities in the primary eye, large drusen in the fellow eye, pigmentary abnormalities in the fellow eye, intermediate drusen in both eyes, late AMD in the fellow eye, and RPD in either eye. These biomarkers were graded using ultra‐widefield retinal photography (Optomap, Daytona, Optos, UK) which is comparable to traditional colour fundus photography for AMD assessment [13, 14, 15]. Late AMD was defined as macular (central or non‐central) geographic atrophy (≥ 175 μm diameter) [16] or neovascularisation [8]. Geographic atrophy was specifically defined using retinal photography to align with AREDS clinical definitions [12]. While other imaging modalities such as OCT‐based criteria can offer more precise structural delineation (e.g., defined as complete retinal pigment epithelium and outer retinal atrophy), these were not used to maintain applicability to clinical risk scale assessment [17]. Neovascularisation was initially confirmed with intravenous angiography [18] and OCT.

RPD was defined using 512 × 128 OCT macular B‐scans (Cirrus HD‐OCT 6000, Carl Zeiss Meditec, Germany). Biomarker definitions followed common literary definitions: large drusen were ‘…> 125 μm in the smallest diameter…’ [12]; intermediate drusen were ‘≥ 63–125 μm (diameter)’ [12]; pigmentary abnormalities were ‘Hyperpigmentation or hypopigmentation…in eyes with drusen 63 μm or more in diameter and without known retinal disease entities or other reasons for such abnormalities” [12]; and RPD were “≥ 5 definite (drusen‐like) lesions seen on > 1 OCT B‐scan…’ above the retinal pigment epithelium [19]. The latter definition for RPD was used to establish a lower threshold for its presence, in contrast to other definitions that are qualitative and somewhat imprecise, such as describing RPD as ‘…clusters of discrete round or oval lesions…’ [8, 20].

Initial diagnosis of early/intermediate AMD was formed by a senior retinal ophthalmologist (SC). Independent gradings of the biomarkers were performed by optometrists (MT and AD). After two rounds/iterations, discrepancies were resolved by the senior retinal ophthalmologist (SC) using the same grading criteria. If any ambiguity remained regarding AMD staging after biomarker grading, the case underwent re‐review by the ophthalmologist to ensure consistency with the Beckman classification. All graders were blinded to the fellow eye and the event of interest, i.e., conversion to late AMD. Grading protocol were defined a priori and followed a trinary certainty system in accordance with AREDS grading [21], categorising the presence of biomarkers as ≥ 90% certainty, 50%–90% certainty, or < 50% certainty. To maintain a high threshold of certainty, a biomarker was considered present only when categorised with ≥ 90% certainty and majority agreement.

### 2.4 Statistical Analysis

Statistical analyses were performed using Prism 10.0.3 (GraphPad, San Diego, CA, USA), Excel 2407 (Microsoft Corporation, Redmond, WA, USA), and Python 3.12.6 (Python Software Foundation, Beaverton, OR, USA). Default statistical significance was *p* < 0.05. Continuous values were presented as mean (95% CI) unless otherwise stated. Inter‐grader agreement was assessed using free‐marginal κ, calculated between the two optometrists [22]. Cumulative risks and adjusted hazard ratios were calculated using Cox proportional hazards regression, with the event of interest being conversion to late AMD. Adjustments for hazard ratios were solely imaging‐based, in line with other AREDS studies calculating clinical risk scores [7, 8]. Participant data were censored at the point of conversion to late AMD or after their last visit within the study period. Comparisons of prognostic performance (area under the receiver operating characteristic curve [AUC]) at the end of each year only included participants with the minimum corresponding follow‐up time and were performed using a *Z*‐score equivalent of Delong's method via Python [23]. Kaplan–Meier risk score curves were assessed for separability using the log‐rank test [24] and reported as the *χ* ^2^ statistic, with values exceeding 3.84 considered significant per comparison (1‐degree of freedom) [25].

## 3 Results

### 3.1 Study Population

A total of 269 participants were consecutively sampled at baseline with an average age of 77.68 (76.76, 78.61) years, 3.17 years follow‐up extending up to 6.94 years, and 57.25% being female (Table 1). There was no significant difference in median follow‐up times for early versus intermediate AMD eyes.

**TABLE 1:** Participant characteristics at baseline.

Characteristic	Value
Age (years)	
Follow‐up (years)	77.68 (76.76, 78.61) 3.17 (up to 6.94 years)
Sex (female)	57.25%
AMD severity (1st eye)	%
Early	15.35
Intermediate	84.65
AMD severity (2nd eye)	%
Late	21.93
Updated simplified AREDS risk scores	%
1 – RPD	11.79
2 – RPD	16.41
3 – RPD	10.77
4 – RPD	9.74
1 + RPD	6.15
2 + RPD	8.21
3 + RPD	17.95
4 + RPD	18.97

The distribution of AMD severity in the primary eye was 15.35% early and 84.65% intermediate stage AMD. In the fellow eye, 21.93% had late AMD. Across both eyes, the distribution of risk according to each updated simplified AREDS risk score ranged between 6.15% and 18.97% (Table 1). Initial inter‐grader agreement was moderate‐to‐strong—89.22% for large drusen (κ, 0.78 [0.71, 0.86]), 83.27% for pigmentary abnormalities (0.65 [0.56, 0.74]), and 94.8% for RPD (0.9 [0.84, 0.95]).

**FIGURE 2:**
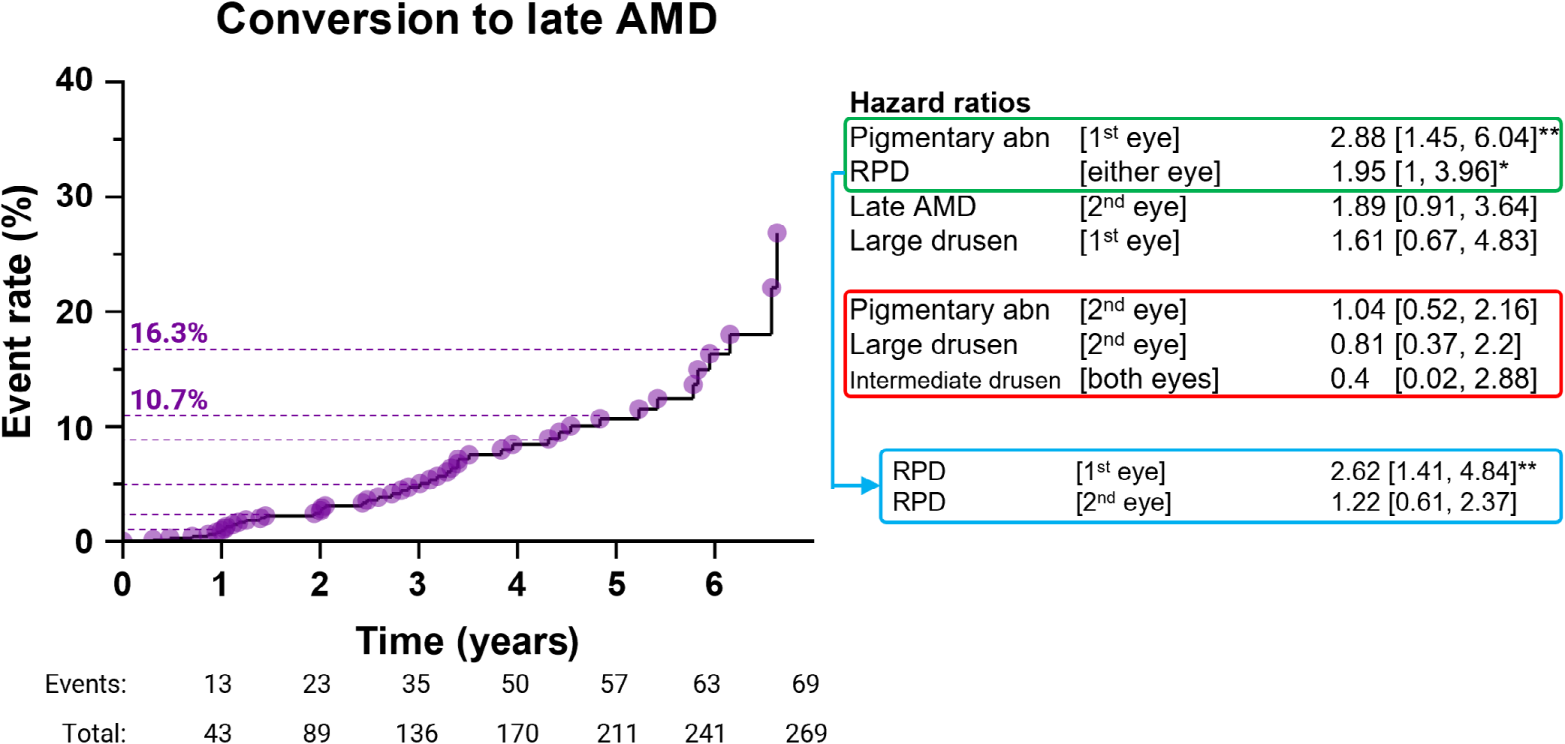
Risk of conversion to late AMD modelled with the updated simplified AREDS risk model. Left: the cumulative risk of conversion to late AMD reached 26.9% over 7 years. At 1 year, the risk for conversion was 1% and subsequently increased to 2.4%, 4.7%, 8.4%, 10.7%, and 16.3% for each year up to 6 years (left, dashed purple lines). The number of patients at risk and cumulative events at each year are shown beneath the *x*‐axis. Right: applying the updated simplified AREDS risk model revealed adjusted hazard ratios where pigmentary abnormalities (1st eye) and RPD (either eye) were the most predictive/significant biomarkers (right in descending order; green outline). Alternatively, pigmentary abnormalities (2nd eye), large drusen (2nd eye), and intermediate drusen (both eyes) were the three least predictive/significant biomarkers in the model (red outline; *p* = 0.43–0.92). When redefining RPD presence as eye‐specific, only RPD in the primary (1st) eye remained predictive (blue outline). *p < 0.05, **p < 0.01; abn, abnormalities.

The total cumulative risk of conversion to late AMD was 26.9% over 7‐years. At 1‐year, the risk for conversion was 1% and subsequently increased to 2.4%, 4.7%, 8.4%, 10.7%, and 16.3% for each year up to 6‐years (Figure 2, left).

### 3.2 Generating Eye‐Level Candidate Risk Models

Applying the full, updated simplified AREDS risk model revealed varying adjusted hazard ratios across biomarkers. The strongest predictors were pigmentary abnormalities in the primary (1st) eye (hazard ratio, 2.88 [1.45, 6.04], *p* < 0.01) and RPD in either eye (1.95 [1, 3.96], *p* < 0.05; Figure 2, right, green outline).

The least predictive biomarkers were pigmentary abnormalities in the fellow (2nd) eye (1.04 [0.52, 2.16], *p* = 0.92), large drusen in the fellow eye (0.81 [0.37, 2.2], *p* = 0.63), and intermediate drusen in both eyes (0.4 [0.02, 2.88], *p* = 0.43; Figure 2, right, red outline).

When redefining RPD presence as eye‐specific, RPD in the primary eye remained predictive (2.62 [1.41, 4.84], *p* < 0.01), whereas RPD in the fellow eye was not (1.22 [0.61, 2.37], *p* = 0.56; Figure 2, right, blue outline).

**FIGURE 3:**
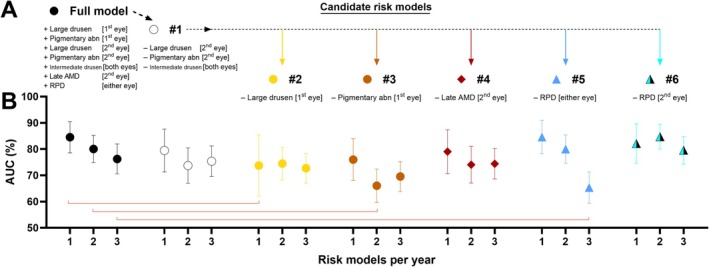
Risk models and prognostic performance. (A) Candidate models were developed via stepwise biomarker removal or modification from the full updated simplified AREDS model (filled circle). Candidate models included: #1 (open circle) which removed large drusen in the fellow eye, pigmentary abnormalities in the fellow eye, and intermediate drusen in both eyes; #2 (yellow circle) which removed large drusen in the primary eye from #1; #3 (brown circle) which removed pigmentary abnormalities in the primary eye from #1; #4 (maroon diamond) which removed late AMD in the fellow eye from #1; #5 (blue triangle) which removed RPD in either eye from #1; and #6 (cyan half‐triangle) which replaced RPD in either eye with RPD in the primary eye only from #1. (B) Prognostic performance (AUC ± SE%) for each risk model per year was then compared to the full model. Exact values provided in Table S1. Models #2, #3, and #5 demonstrated reduced prognostic performance at 1‐, 2‐, and 3‐year intervals, respectively (*p* < 0.05, red lines). No significant differences were observed at 4‐, 5‐, and 6‐year intervals. Abn, abnormalities.

Based on these findings, several abridged candidate risk models were developed by stepwise removal or reconfiguration of biomarkers from the full, updated simplified AREDS risk model (Figure 3A). Specifically, the candidate risk models were:

- #1 (Figure 3A, open circle), which removed the three least predictive variables (large drusen and pigmentary abnormalities in the fellow eye, and intermediate drusen in both eyes).
- #2 (Figure 3A, yellow circle), which removed large drusen in the primary eye from model #1.
- #3 (Figure 3A, brown circle), which removed pigmentary abnormalities in the primary eye from #1.
- #4 (Figure 3A, red diamond), which removed late AMD in the fellow eye from model #1.
- #5 (Figure 3A, blue triangle), which removed RPD in either eye from model #1.
- #6 (Figure 3A, cyan half‐triangle), which replaced RPD in either eye with RPD in the primary eye only from #1.

### 3.3 Prognostic Performance

The prognostic performance of each eye‐level candidate risk model was compared to the full person‐level updated simplified AREDS risk model, the latter yielding AUCs between 76.24% ± 5.72% and 84.52% ± 5.93% at 1‐ to 3‐years (Figure 3A, filled circle; Table S1).

Candidate risk model #1 showed comparable AUCs (73.72% ± 6.74% to 79.45% ± 8.17%; Figure 3B, open circles) and was not significantly different from the full model (*p* ≥ 0.25).

In contrast, reduced performance was observed for models:

- #2 at 1‐year (73.73% ± 11.68%, *p* < 0.05; Figure 3B, yellow circles).
- #3 at 2‐years (66.06% ± 6.36%, *p* < 0.05; Figure 3B, brown circles).
- #5 at 3‐years (65.33% ± 5.92%, *p* < 0.05; Figure 3B, blue triangles).

Models #4 and #6 both maintained similar AUCs (74.04% ± 6.98% to 84.71% ± 4.72%) and were not significantly different from the full model at any year.

No candidate risk models were significantly different from the full model at 4‐, 5‐, and 6‐years. In summary, candidate risk models #1, #4, and #6 maintained similar prognostic performance to the full, updated simplified AREDS risk model.

### 3.4 Separability of Risk Scores

**FIGURE 4:**
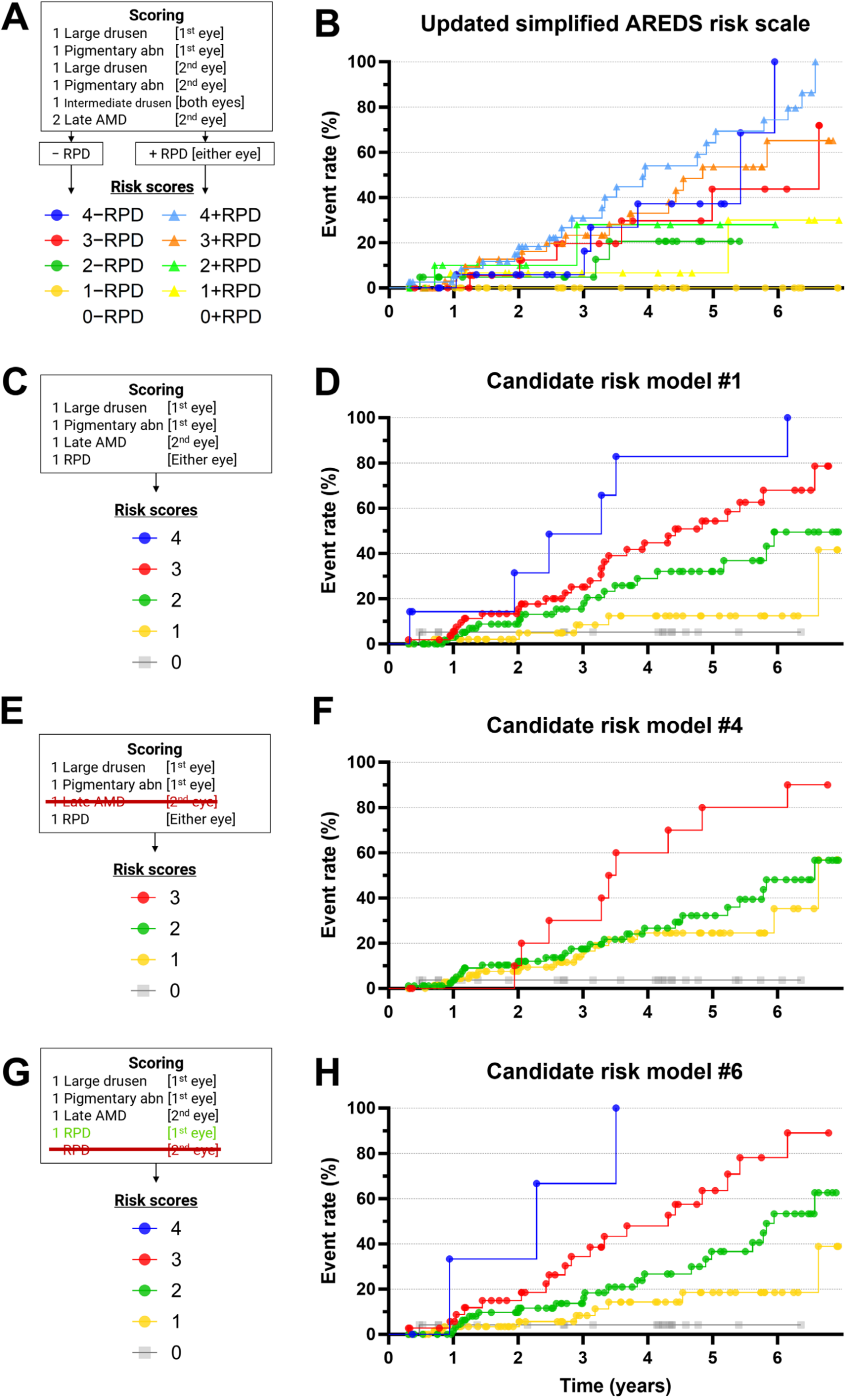
Risk models' scoring and curves. Left: the risk model scoring system, and right: its corresponding cumulative risk score curves for conversion to late AMD. These are displayed for the (A, B) full, updated simplified AREDS risk scale, (C, D) model #1, (E, F) model #4, and (G, H) model #6, as defined in Figure 3A. Exact values provided in Table S3. No participants had an updated simplified AREDS risk score of 0 (with or without RPD).

Subsequently, separability of risk scores was evaluated for candidate models #1, #4, and #6, alongside the full, updated simplified AREDS model. The full model exhibited substantial overlap between adjacent risk score curves (Figure 4A,B), with 5 out of 6 comparisons showing no significant difference (*χ* ^2^ ≤ 2.08, *p* > 0.05; Table 2 red cells). Moreover, overlap was also common (13/22) for non‐adjacent risk scores (e.g., score 2 − RPD vs. 4 − RPD, or 3 − RPD vs. 1 + RPD), which are supposed to be even more distinct (Table S2). Overlaps between risk scores were similar to the original population of the updated simplified AREDS risk model scores [8].

**TABLE 2:** Separability between adjacent risk score curves.

				Adjacent risk score comparisons			
Risk model	Biomarkers			0 vs. 1	1 vs. 2	2 vs. 3	3 vs. 4
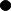 Full	+Large drusen +Pigmentary abn +Large drusen +Pigmentary abn +Intermediate drusen +Late AMD	(1st eye) (1st eye) (2nd eye) (2nd eye) (both eyes) (2nd eye)	−RPD	—	3.9*	0.49	0.52
			+RPD (either eye)	—	0.56	0.46	2.08
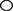 Candidate #1	−Large drusen −Pigmentary abn −Intermediate drusen	(2nd eye) (2nd eye) (both eyes)		0.16	3.87*	3.85*	3.93*
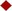 Candidate #4	−Late AMD	(2nd eye)		3.1	0.69	6.67**	—
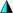 Candidate #6	+RPD −RPD	(1st eye) (2nd eye)		0.77	4.06*	7.74**	4.28*

*Note*: Separability values represented by the *χ* ^2^ statistic for adjacent risk score curves, where a higher value denotes greater separability. *χ* ^2^ > 3.84 is considered significant per comparison (1‐degree of freedom). Shaded in light red are non‐separable adjacent risk scores. *p < 0.05 and ***p < 0.001; vs., versus.

For each candidate model, a score of ‘1’ per biomarker was assigned due to comparable associated risks (adjusted hazard ratios, Figure 2, right).

- Model #1 showed improved separation (Figure 4C,D), with three out of four adjacent score comparisons reaching significance (χ^2^ ≥ 3.85, *p* < 0.05; Table 2 white cells).
- Model #4 showed less separation (Figure 4E,F), with only one out of three adjacent score comparisons reaching significance (*χ* ^2^ = 6.67, *p* < 0.01; Table 2 white cells).
- Model #6 showed improved separation (Figure 4G,H), with three out of four adjacent score comparisons reaching significance (*χ* ^2^ ≥ 4.06. *p* < 0.05; Table 2 white cells).

In summary, models #1 and #6 provided more distinct risk separation compared to the full model.

### 3.5 An Example Application of the Simpler Risk Scores

Both candidate risk model #1 and #6 demonstrated similar prognostic performance and improved separability of risk score curves relative to the full, updated simplified AREDS risk model.

**FIGURE 5:**
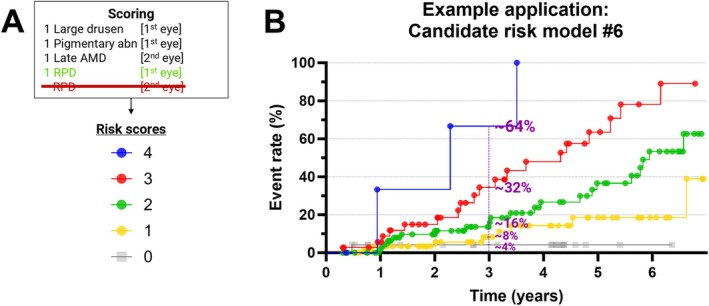
An example of 3‐year simpler risk scores. (A) The risk scoring model system and its (B) corresponding cumulative risk score curves for conversion to late AMD, for model #6. The projected 3‐year risks (scores 0–4) were 4.2%, 8.3%, 13.6%, 34.5%, and 66.7%. Exact values provided in Table S3. For ease‐of‐interpretation, these could be approximated to ≈4%, 8%, 16%, 32%, and 64%.

As model #6 had a lesser total number of observations, it was selected as the final proposed model and used to provide an example application of 3‐year simpler risk scores (Figure 5A,B). The projected 3‐year risks (scores 0–4) were 4.2%, 8.3%, 13.6%, 34.5%, and 66.7% (Table S3). For ease‐of‐interpretation, these could be approximated to ≈4%, 8%, 16%, 32%, and 64%.

## 4 Discussion

This study demonstrates that the updated simplified AREDS risk model can be essentially halved without compromising prognostic performance by (1) deriving eye‐specific biomarkers and (2) assigning one point per biomarker (large drusen, pigmentary abnormalities, and RPD in the primary eye, and late AMD in the fellow eye). An abridged, eye‐level risk scale is proposed that may enhance clinical efficiency when one AMD eye is of more interest than the other. It also extends the applicability of the AREDS model to support inter‐eye risk‐adjusted study designs. An example of 3‐year risks (scores 0–4) was ≈4%, 8%, 16%, 32%, and 64%. These preliminary results warrant further validation in larger populations with extended follow‐up.

### 4.1 Corroborating the Inclusion of RPD Into AMD Prognostication

The proposed modifications streamline use of the simplified AREDS risk models [7, 8], further leveraging RPD as a key prognostic biomarker for AMD progression and partially validating its use in a real‐world clinical population. While other OCT biomarkers, such as outer retinal band disruptions, are associated with even higher risks of AMD progression relative to RPD [26, 27], their integration would necessitate more reliable, automated, and quantitative processing techniques due to the difficulty/non‐reliability of manual detection [28]. Thus, this study focused on manually graded RPD using OCT, which is becoming increasingly accessible [29]. Moreover, these findings are not necessarily limited to OCT‐defined RPD; rather, OCT was selected as the most sensitive imaging modality for RPD [30], corroborated by the excellent inter‐grader reliability in this study. Results may also be translatable to other imaging modalities, such as retinal photography as used in the original AREDS populations [8], albeit with a possible reduction in prognostic performance due to lower sensitivity of the imaging modality.

While the prognostic performance of the full person‐level updated simplified AREDS and eye‐level simpler models were fair [31], they do likely under‐perform relative to other more complex models in the literature demonstrating AUCs approaching 90% or higher [32, 33, 34]. However, the main strength of the simplified AREDS models lies in their accessibility. Unlike other models that require complex assessments such as artificial intelligence algorithms, custom image processing, or genetic testing, the simplified AREDS models and its potential reconfigurations only use readily available, manually interpretable retinal imaging data, thereby minimising the need for additional measures and reducing clinical time and/or computational demands. Additionally, they avoid reliance on factors that may be inconsistently reported, such as race/ethnicity, or those subject to recall bias, such as pharmaceutical drug use [35].

### 4.2 Comparison to the AREDS Population

There were some differences between this study's contained, open‐cohort population and the AREDS much larger‐ and longer‐scale cohort, particularly regarding the prevalence of RPD which was significantly higher in this study (≈30% in a single eye and ≈50% in either eye, vs. < 10% in the AREDS cohort) [8, 36]. This was consistent with findings from other studies using OCT [37, 38], demonstrating more sensitive detection of RPD using OCT compared to colour fundus photography [30]. The higher prevalence in this study can also be attributed to the older average age of participants (78 vs. 69 years in the AREDS cohort) [8] and a higher proportion of intermediate AMD versus early AMD, both of which are associated with an increased prevalence of RPD [30]. Despite these differences, most of our person‐level risks aligned with those published by Agrón et al., bolstering generalisability of the AREDS risk model and our proposed eye‐level re‐configurations, the latter pending further larger‐scale validation to account for our limited sampling [8].

### 4.3 Limitations and Future Directions

The primary limitation of this study is its retrospective design and relatively contained population, which may introduce ascertainment bias and limit further sub‐analyses such as stratifying risk of conversion to neovascular versus atrophic late AMD. Additionally, race and ethnicity data were not consistently captured in this real‐world clinical setting and were therefore unavailable for analysis. As these factors may influence AMD prevalence and progression, this limits generalisability and will be addressed in future prospective cohorts. Furthermore, this study population was drawn exclusively from a metropolitan region of Sydney, Australia, which may not be representative of rural settings or international healthcare systems. Broader validation across demographically and geographically diverse populations is needed to support the external applicability of the proposed model.

The open‐cohort design also helps explain the underrepresentation of early AMD (15% of study eyes) relative to intermediate AMD (85%), as patients with early AMD are less likely to be referred to tertiary eye care centres. However, this imbalance only affected the depiction of the updated simplified AREDS cumulative risk for score 0 (representing early AMD in a single eye) and did not impact any overarching conclusions. Further validation is warranted to refine the optimal reconfiguration of the risk scale, particularly in larger cohorts with extended follow‐up, which may also support downstream clinical impact analyses, such as decision‐curve modelling [39].

Importantly, this study does not propose a new model as others have done using OCT biomarkers [40]. Instead, it provides evidence that abridged eye‐level configurations of the updated simplified AREDS risk scale may improve workflow efficiency and enable inter‐eye risk calculations in future studies. The proposed re‐configurations assume that RPD, large drusen, and pigmentary abnormalities in the primary eye, along with late AMD in the fellow eye, contribute similar risks and hence risk scores. While this may seem unintuitive, particularly since large drusen is a defining feature of AMD [12, 41], a high‐risk diagnostic biomarker does not necessarily equate to strong prognostic performance, which depends on the ability to discriminate and calibrate the likelihood of an event (conversion to late AMD) [42]. Given the near‐ubiquitous presence of large drusen in intermediate AMD by definition, consequently, it may not be a significantly stronger predictor of progression than other biomarkers once considered concurrently. This aligns with studies demonstrating that AMD biomarkers are largely collinear, and their co‐presence can significantly influence risk. This supports our decision to assign equal weighting based on reasonably similar adjusted hazard ratios. However, these scores are not intended as a definitive guide for re‐configuring the AREDS risk scale, but rather serve as potential options for streamlining risk assessments.

Finally, the sample size of 269 patients reflected the number of eligible cases identified over a fixed 7‐year sampling window. While modest, this cohort allowed for meaningful modelling of short‐ to mid‐term outcomes. Given the observed follow‐up distribution, prognostic estimates were primarily based on three‐year data, which corresponded to the cohort's mean follow‐up duration. Future studies will aim to expand the sample size and extend follow‐up periods to improve long‐term risk prediction.

### 4.4 Conclusion

The updated simplified AREDS risk model can be essentially halved without compromising prognostic performance by (1) deriving eye‐specific biomarkers and (2) assigning one point per biomarker (large drusen, pigmentary abnormalities, and RPD in the primary eye, and late AMD in the fellow eye). This eye‐level risk stratification may improve clinical efficiency when one AMD eye is of more interest than the other. It also extends the applicability of the AREDS model to support inter‐eye risk‐adjusted study designs. An example of 3‐year risks (scores 0–4) was ≈4%, 8%, 16%, 32%, and 64%. These preliminary results warrant further validation in larger populations with extended follow‐up.

## Conflicts of Interest

The authors declare no conflicts of interest.

## Supporting information


**Data S1.** Supporting Information.

## Data Availability

The data that support the findings of this study are available on request from the corresponding author. The data are not publicly available due to privacy or ethical restrictions.

## References

[1] J. Mitchell and C. Bradley , “Quality of Life in Age‐Related Macular Degeneration: A Review of the Literature,” Health and Quality of Life Outcomes 4 (2006): 97. 17184527 10.1186/1477-7525-4-97 PMC1780057

[2] C. Hopley , G. Salkeld , J. J. Wang , and P. Mitchell , “Cost Utility of Screening and Treatment for Early Age Related Macular Degeneration With Zinc and Antioxidants,” British Journal of Ophthalmology 88 (2004): 450–454. 15031152 10.1136/bjo.2003.035279 PMC1772079

[3] C. J. Flaxel , R. A. Adelman , S. T. Bailey , et al., “Age‐Related Macular Degeneration Preferred Practice Pattern,” Ophthalmology 127 (2020): P1–P65. 31757502 10.1016/j.ophtha.2019.09.024

[4] U. Chakravarthy and M. Williams , “The Royal College of Ophthalmologists Guidelines on AMD: Executive Summary,” Eye (Lond) 27 (2013): 1429–1431. 10.1038/eye.2013.233 PMC3869519 24158023

[5] K. M. Hart , C. Abbott , A. Ly , et al., “Optometry Australia's Chairside Reference for the Diagnosis and Management of Age‐Related Macular Degeneration,” Clinical & Experimental Optometry 103 (2020): 254–264. 10.1111/cxo.12964 31566818

[6] R. S. Apte , “Age‐Related Macular Degeneration,” New England Journal of Medicine 385 (2021): 539–547. 10.1056/NEJMcp2102061 PMC9369215 34347954

[7] F. L. Ferris , M. D. Davis , T. E. Clemons , et al., “A Simplified Severity Scale for Age‐Related Macular Degeneration: AREDS Report No. 18,” Archives of Ophthalmology 123 (2005): 1570–1574. 16286620 10.1001/archopht.123.11.1570 PMC1473206

[8] E. Agrón , A. Domalpally , Q. Chen , et al., “An Updated Simplified Severity Scale for Age‐Related Macular Degeneration Incorporating Reticular Pseudodrusen: Age‐Related Eye Disease Study Report Number 42,” Ophthalmology 131 (2024): 1164–1174. 10.1016/j.ophtha.2024.04.011 PMC11416341 38657840

[9] O. Trivizki , L. Wang , Y. Shi , et al., “Symmetry of Macular Fundus Features in Age‐Related Macular Degeneration,” Ophthalmology Retina 7 (2023): 672–682. 37003480 10.1016/j.oret.2023.03.016 PMC10614575

[10] P. Pallmann , A. W. Bedding , B. Choodari‐Oskooei , et al., “Adaptive Designs in Clinical Trials: Why Use Them, and How to Run and Report Them,” BMC Medicine 16 (2018): 29. 29490655 10.1186/s12916-018-1017-7 PMC5830330

[11] RANZCO , “RANZCO: Referral Pathway for AMD Management,” 2024.

[12] F. L. Ferris , C. P. Wilkinson , A. Bird , et al., “Clinical Classification of Age‐Related Macular Degeneration,” Ophthalmology 120 (2013): 844–851. 23332590 10.1016/j.ophtha.2012.10.036 PMC11551519

[13] A. Csutak , I. Lengyel , F. Jonasson , et al., “Agreement Between Image Grading of Conventional (45°) and Ultra Wide‐Angle (200°) Digital Images in the Macula in the Reykjavik Eye Study,” Eye 24 (2010): 1568–1575. 10.1038/eye.2010.85 20523357

[14] M. Maruyama‐Inoue , Y. Kitajima , S. Mohamed , et al., “Sensitivity and Specificity of High‐Resolution Wide Field Fundus Imaging for Detecting Neovascular Age‐Related Macular Degeneration,” PLoS One 15 (2020): e0238072. 10.1371/journal.pone.0238072 PMC7442256 32822418

[15] P. Ramtohul , P. Gascon , A. Comet , and D. Denis , “Ultrawidefield Pseudocolor Retinal Imaging Versus Real‐Color Fundus Photography for Detection of Intraretinal Pigment Migration in Age‐Related Macular Degeneration,” Retina 41 (2021): 563–571. 33600133 10.1097/IAE.0000000000002886

[16] S. Fraser‐Bell , F. Choudhury , R. Klein , S. Azen , and R. Varma , “Ocular Risk Factors for Age‐Related Macular Degeneration: The Los Angeles Latino Eye Study (LALES),” American Journal of Ophthalmology 149 (2010): 735–740. 20138605 10.1016/j.ajo.2009.11.013 PMC2856762

[17] R. H. Guymer , P. J. Rosenfeld , C. A. Curcio , et al., “Incomplete Retinal Pigment Epithelial and Outer Retinal Atrophy in Age‐Related Macular Degeneration: Classification of Atrophy Meeting Report 4,” Ophthalmology 127 (2020): 394–409. 31708275 10.1016/j.ophtha.2019.09.035 PMC7218279

[18] RANZCO , “RANZCO: Fluorescein and Indocyanine Green Angiography Guidelines,” 2023.

[19] Z. Wu , H. Kumar , L. A. B. Hodgson , and R. H. Guymer , “Reticular Pseudodrusen on the Risk of Progression in Intermediate Age‐Related Macular Degeneration,” American Journal of Ophthalmology 239 (2022): 202–211. 10.1016/j.ajo.2022.03.007 35288077

[20] Q. Chen , T. D. Keenan , A. Allot , et al., “Multimodal, Multitask, Multiattention (M3) Deep Learning Detection of Reticular Pseudodrusen: Toward Automated and Accessible Classification of Age‐Related Macular Degeneration,” Journal of the American Medical Informatics Association 28 (2021): 1135–1148. 10.1093/jamia/ocaa302 PMC8200273 33792724

[21] Age‐Related Eye Disease Study Research Group , “The Age‐Related Eye Disease Study System for Classifying Age‐Related Macular Degeneration From Stereoscopic Color Fundus Photographs: The Age‐Related Eye Disease Study Report Number 6,” American Journal of Ophthalmology 132 (2001): 668–681. 11704028 10.1016/s0002-9394(01)01218-1

[22] J. J. Randolph , “Free‐Marginal Multirater Kappa (Multirater k[Free]): An Alternative to Fleiss' Fixed‐Marginal Multirater Kappa,” 2005, https://eric.ed.gov/?id=ED490661.

[23] X. Sun and W. Xu , “Fast Implementation of Delong's Algorithm for Comparing the Areas Under Correlated Receiver Operating Characteristic Curves,” IEEE Signal Processing Letters 21 (2014): 1389–1393.

[24] J. M. Bland and D. G. Altman , “The Logrank Test,” BMJ 328 (2004): 1073. 10.1136/bmj.328.7447.1073 PMC403858 15117797

[25] H.‐Y. Kim , “Statistical Notes for Clinical Researchers: Chi‐Squared Test and Fisher's Exact Test,” Restorative Dentistry and Endodontics 42 (2017): 152–155. 28503482 10.5395/rde.2017.42.2.152 PMC5426219

[26] M. Trinh , R. Cheung , A. Duong , L. Nivison‐Smith , and A. Ly , “OCT Prognostic Biomarkers for Progression to Late Age‐Related Macular Degeneration: A Systematic Review and Meta‐Analysis,” Ophthalmology Retina 8 (2024): 553–565, 10.1016/j.oret.2023.12.006. 38154619

[27] M. Trinh , R. Cheung , J. Nam , L. Nivison‐Smith , and A. Ly , “Improving Prognostic Discrimination of Late AMD Using Four OCT Biomarkers,” Investigative Ophthalmology & Visual Science 65 (2024): 4377.

[28] J. Nam , M. Trinh , R. Cheung , A. Duong , and L. Nivison‐Smith , “Four OCT Prognostic Biomarkers for Age‐Related Macular Degeneration (AMD) Achieve Higher Inter‐Grader Agreement Than the Gold‐Standard Large Drusen and/or Pigmentary Abnormalities,” Investigative Ophthalmology & Visual Science 65 (2024): 1433.

[29] R. Cheung , S. Ho , and A. Ly , “Optometrists' Attitudes Toward Using OCT Angiography Lag Behind Other Retinal Imaging Types,” Ophthalmic and Physiological Optics 43 (2023): 905–915. 37082888 10.1111/opo.13149

[30] Z. Wu , E. L. Fletcher , H. Kumar , U. Greferath , and R. H. Guymer , “Reticular Pseudodrusen: A Critical Phenotype in Age‐Related Macular Degeneration,” Progress in Retinal and Eye Research 88 (2021): 101017. 34752916 10.1016/j.preteyeres.2021.101017

[31] S. Safari , A. Baratloo , M. Elfil , and A. Negida , “Evidence Based Emergency Medicine; Part 5 Receiver Operating Curve and Area Under the Curve,” Emergency 4 (2016): 111–113. PMC4893763 27274525

[32] K. Sarici , J. R. Abraham , D. D. Sevgi , et al., “Risk Classification for Progression to Subfoveal Geographic Atrophy in Dry Age‐Related Macular Degeneration Using Machine Learning‐Enabled Outer Retinal Feature Extraction,” Ophthalmic Surgery, Lasers & Imaging Retina 53 (2022): 31–39. 10.3928/23258160-20211210-01 34982004

[33] Z. Wu , H. Bogunović , R. Asgari , U. Schmidt‐Erfurth , and R. H. Guymer , “Predicting Progression of Age‐Related Macular Degeneration Using OCT and Fundus Photography,” Ophthalmology Retina 5 (2021): 118–125. 32599175 10.1016/j.oret.2020.06.026

[34] E. R. Dow , H. K. Jeong , E. A. Katz , et al., “A Deep‐Learning Algorithm to Predict Short‐Term Progression to Geographic Atrophy on Spectral‐Domain Optical Coherence Tomography,” JAMA Ophthalmology 141 (2023): 1052–1061. 37856139 10.1001/jamaophthalmol.2023.4659 PMC10587827

[35] R. Kehoe , S. Y. Wu , M. C. Leske , and L. T. Chylack , “Comparing Self‐Reported and Physician‐Reported Medical History,” American Journal of Epidemiology 139 (1994): 813–818. 10.1093/oxfordjournals.aje.a117078 8178794

[36] E. Agrón , A. Domalpally , C. A. Cukras , et al., “Reticular Pseudodrusen: The Third Macular Risk Feature for Progression to Late Age‐Related Macular Degeneration: Age‐Related Eye Disease Study 2 Report 30,” Ophthalmology 129 (2022): 1107–1119. 10.1016/j.ophtha.2022.05.021 PMC9509418 35660417

[37] H. Chan , A. Cougnard‐Grégoire , M. N. Delyfer , et al., “Multimodal Imaging of Reticular Pseudodrusen in a Population‐Based Setting: The ALIENOR Study,” Investigative Ophthalmology & Visual Science 57 (2016): 3058–3065. 10.1167/iovs.16-19487 27367498

[38] P.‐H. Gabrielle , A. Seydou , L. Arnould , et al., “Subretinal Drusenoid Deposits in the Elderly in a Population‐Based Study (The Montrachet Study),” Investigative Ophthalmology & Visual Science 60 (2019): 4838–4848. 31747683 10.1167/iovs.19-27283

[39] M. Assel , D. D. Sjoberg , and A. J. Vickers , “The Brier Score Does Not Evaluate the Clinical Utility of Diagnostic Tests or Prediction Models,” Diagnostic and Prognostic Research 1 (2017): 19. 10.1186/s41512-017-0020-3 PMC6460786 31093548

[40] J. Lei , S. Balasubramanian , N. S. Abdelfattah , M. G. Nittala , and S. R. Sadda , “Proposal of a Simple Optical Coherence Tomography‐Based Scoring System for Progression of Age‐Related Macular Degeneration,” Graefe's Archive for Clinical and Experimental Ophthalmology 255 (2017): 1551–1558. 10.1007/s00417-017-3693-y 28534244

[41] R. Klein , B. E. K. Klein , M. D. Knudtson , S. M. Meuer , M. Swift , and R. E. Gangnon , “Fifteen‐Year Cumulative Incidence of Age‐Related Macular Degeneration: The Beaver Dam Eye Study,” Ophthalmology 114 (2007): 253–262. 10.1016/j.ophtha.2006.10.040 17270675

[42] A. C. Alba , T. Agoritsas , M. Walsh , et al., “Discrimination and Calibration of Clinical Prediction Models: Users' Guides to the Medical Literature,” JAMA 318 (2017): 1377–1384. 29049590 10.1001/jama.2017.12126

