# Supplementary material for: Proposal of a Simpler Eye‐Level Risk Model Incorporating Reticular Pseudodrusen for the Clinical Prediction of Late Age‐Related Macular Degeneration
Source: Clin Exp Ophthalmol. 2025 Jul 3;53(8):936–45. doi: 10.1111/ceo.14576 (PMC12596412; doi:10.1111/ceo.14576)
Supplement: Supplementary file 1 — Data S1. Supporting Information. [file CEO-53-936-s001.docx]

# Supplementary Tables

Proposal of a Simpler Eye-Level Risk Model

Incorporating Reticular Pseudodrusen

for the Clinical Prediction of Late AMD

Matt Trinh (PhD),*^1^* Annita Duong,*^1^* Rene Cheung,*^1^* Simon Chen (FRANZCO),*^1^*^,^*^2^* David Ng (FRANZCO),*^2^* Jeff Friedrich (FRANZCO),*^2^* Chris Hodge (PhD),*^2^* Lisa Nivison-Smith (PhD),*^1^* Angelica Ly (PhD)*^1^*

*^1^ School of Optometry and Vision Science, University of New South Wales, Sydney, NSW, Australia*

*^2^ Vision Eye Institute, Sydney, NSW, Australia*

**Correspondence:** Dr Matt Trinh; [m.trinh@unsw.edu.au](mailto:m.trinh@unsw.edu.au)

School of Optometry and Vision Science, UNSW Sydney, 2015, NSW, Australia.

**S Table 1. Prognostic performance of candidate simpler risk models.**

Prognostic performance expressed as AUC % ± SE, corresponding to *Figure 4*. Candidate risk models #2, #3, and #5 demonstrated reduced prognostic performance at 1-, 2-, and 3-years respectively (highlighted in *red*), relative to the full model. No candidate risk models were significantly different to the full model at 4-, 5-, and 6-years.

|  | | **Years** | | | | | |
| --- | --- | --- | --- | --- | --- | --- | --- |
| **Risk model** | **Biomarkers** | **1** | **2** | **3** | **4** | **5** | **6** |
| **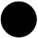 Full** | +Large drusen [1^st^ eye]  +Pigmentary abn [1^st^ eye]  +Large drusen [2^nd^ eye]  +Pigmentary abn [2^nd^ eye]  +Intermediate drusen [both eyes]  +Late AMD [2^nd^ eye]  +RPD [either eye] | 84.52 ± 5.93 | 80.04 ± 5.18 | 76.24 ± 5.72 | 75.57 ± 5.52 | 72.85 ± 6.18 | 73.04 ± 8.42 |
| **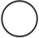 Candidate #1** | −Large drusen [2^nd^ eye]  −Pigmentary abn [2^nd^ eye]  −Intermediate drusen [both eyes] | 79.45 ± 8.17 | 73.72 ± 6.74 | 75.38 ± 5.82 | 75.32 ± 5.31 | 72.49 ± 5.89 | 72.03 ± 7.35 |
| **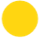 Candidate #2** | −Large drusen [1^st^ eye] | 73.73 ± 11.68 | 74.49 ± 6.23 | 72.7 ± 5.65 | 74.05 ± 5.27 | 69.89 ± 5.96 | 71.91 ± 7.2 |
| **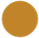 Candidate #3** | −Pigmentary abn [1^st^ eye] | 76.02 ± 7.95 | 66.06 ± 6.36 | 69.54 ± 5.71 | 69.72 ± 5.23 | 67.33 ± 5.99 | 61.36 ± 7.8 |
| **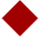 Candidate #4** | −Late AMD [2^nd^ eye] | 79.01 ± 8.32 | 74.04 ± 6.98 | 74.42 ± 5.78 | 74.1 ± 5.11 | 68.94 ± 6.11 | 70.58 ± 7.61 |
| **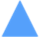 Candidate #5** | −RPD [either eye] | 84.59 ± 6.38 | 80 ± 5.43 | 65.33 ± 5.92 | 75.57 ± 5.49 | 71.75 ± 6.33 | 72.92 ± 6.56 |
| 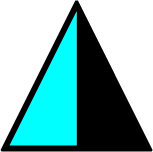 **Candidate #6** | +RPD [1^st^ eye]  −RPD [2^nd^ eye] | 82.08 ± 7.52 | 84.71 ± 4.72 | 79.53 ± 5.26 | 78.76 ± 5.12 | 75.13 ± 5.95 | 79.9 ± 6.37 |

**S Table 2. Separability between all risk score curves in the full, updated simplified AREDS risk model.**

Separability values represented by the χ^2^ statistic for adjacent (*purple outline*; as described in *Table 2*) and non-adjacent risk score curves, where a higher value denotes greater separability. χ^2^ > 3.84 is considered significant per comparison (1-degree of freedom).^1^ *Shaded in light red* are non-separable scores.

* *P* < 0.05 and *** *P* < 0.001.

| **Risk scores** | | **− RPD** | | | | **+ RPD** | | | |
| --- | --- | --- | --- | --- | --- | --- | --- | --- | --- |
|  |  | **1** | **2** | **3** | **4** | **1** | **2** | **3** | **4** |
| **− RPD** | **1** |  | 3.9* | 8.63** | 9.76** | 3.87* | 6.2* | 12.4*** | 22.41*** |
|  | **2** |  |  | 0.49 | 0.65 | 0.14 | 0.26 | 1.8 | 5.69* |
|  | **3** |  |  |  | 0.52 | 1.16 | 0.03 | 0.29 | 5.03* |
|  | **4** |  |  |  |  | 1.61 | 0.08 | 0.2 | 2.05 |
| **+ RPD** | **1** |  |  |  |  |  | 0.56 | 2.82 | 7.97** |
|  | **2** |  |  |  |  |  |  | 0.46 | 2.01 |
|  | **3** |  |  |  |  |  |  |  | 2.08 |
|  | **4** |  |  |  |  |  |  |  |  |

**S Table 3. Risk of conversion to late AMD with various risk models.**

The cumulative risks of conversion to late AMD expressed as percentages (%). These values correspond to *Figure 4*. Candidate risk model #1 removed large drusen in the primary eye, pigmentary abnormalities in the primary eye, and int drusen in both eyes from the full, updated simplified AREDS risk model. Candidate risk model #2 further removed late AMD in the fellow eye from model #1. Candidate risk model #6 added RPD in the primary eye and removed RPD in the fellow eye from model #1.

|  | | | **Years** | | | | | | |
| --- | --- | --- | --- | --- | --- | --- | --- | --- | --- |
|  |  |  | **1** | **2** | **3** | **4** | **5** | **6** | **Total** |
| **Updated simplified**  **AREDS risk model** | **− RPD** | **0** 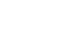 | - | - | - | - | - | - | - |
|  |  | **1** 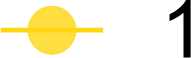 | 0 | 0 | 0 | 0 | 0 | 0 | 0 |
|  |  | **2** **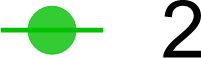** | 4.8 | 4.8 | 4.8 | 20.6 | 20.6 | 20.6 | 20.6 |
|  |  | **3** **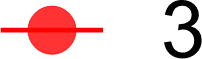** | 0 | 5.6 | 19.6 | 19.6 | 19.6 | 19.6 | 59.8 |
|  |  | **4 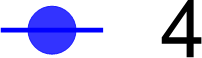** | 0 | 5.9 | 5.9 | 37.3 | 37.3 | 100 | 100 |
|  | **+RPD**  **[either eye]** | **0** 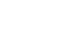 | - | - | - | - | - | - | - |
|  |  | **1** **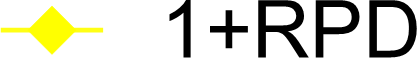** | 6.7 | 6.7 | 6.7 | 6.7 | 6.7 | 30 | 30 |
|  |  | **2** **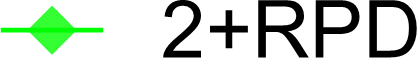** | 10 | 10 | 28 | 28 | 28 | 28 | 28 |
|  |  | **3** **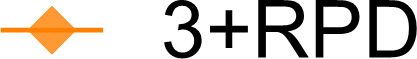** | 3.1 | 12.7 | 23.4 | 28.2 | 50.3 | 62.7 | 62.7 |
|  |  | **4** **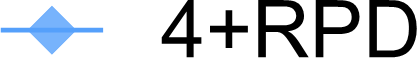** | 5.7 | 15 | 26.9 | 46.8 | 46.8 | 55.7 | 100 |
| **Candidate**  **risk models** | **#1** | **0** **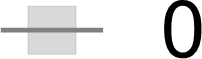** | 5.3 | 5.3 | 5.3 | 5.3 | 5.3 | 5.3 | 5.3 |
|  |  | **1** **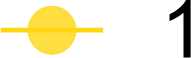** | 2.1 | 2.1 | 4.9 | 4.9 | 11.2 | 11.2 | 11.2 |
|  |  | **2** **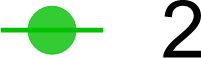** | 1.7 | 10.5 | 21.5 | 34 | 40.4 | 62.4 | 100 |
|  |  | **3** **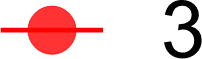** | 5.6 | 13.3 | 25.2 | 44.7 | 54.4 | 68 | 89.3 |
|  |  | **4 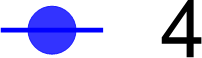** | 14.3 | 31.4 | 48.6 | 82.9 | 82.9 | 82.9 | 100 |
|  | **#4** | **0** **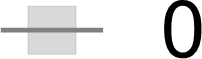** | 4.8 | 4.8 | 4.8 | 4.8 | 4.8 | 4.8 | 4.8 |
|  |  | **1** **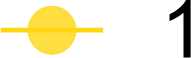** | 2 | 2 | 8.1 | 15.4 | 15.4 | 15.4 | 43.6 |
|  |  | **2** **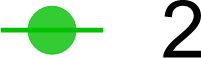** | 1.5 | 7.6 | 11.5 | 23.7 | 36.8 | 54.6 | 54.6 |
|  |  | **3** **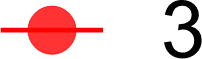** | 6.5 | 18.2 | 32.8 | 47.2 | 47.2 | 59.2 | 83 |
|  | **#6** | **0** **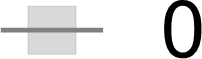** | 4.2 | 4.2 | 4.2 | 4.2 | 4.2 | 4.2 | 4.2 |
|  |  | **1** **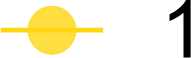** | 3.4 | 3.4 | 8.3 | 14.3 | 18.5 | 18.5 | 38.9 |
|  |  | **2** **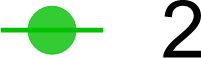** | 1.6 | 9.7 | 13.6 | 26.7 | 36.6 | 53.3 | 62.6 |
|  |  | **3** **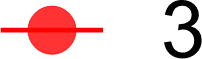** | 5.7 | 15 | 34.5 | 48 | 63.5 | 78.1 | 89.1 |
|  |  | **4 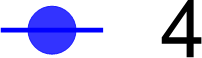** | 33.3 | 33.3 | 66.7 | 100 | 100 | 100 | 100 |
